# Long-read transcriptome sequencing provides insight into lignan biosynthesis during fruit development in *Schisandra chinensis*

**DOI:** 10.1186/s12864-021-08253-2

**Published:** 2022-01-08

**Authors:** Chang Pyo Hong, Chang-Kug Kim, Dong Jin Lee, Hee Jeong Jeong, Yi Lee, Sin-Gi Park, Hyo-Jin Kim, Ji-Nam Kang, Hojin Ryu, Soo-Jin Kwon, Sang-Ho Kang

**Affiliations:** 1 Theragen Bio Co., Ltd., Suwon, 16229 Republic of Korea; 2 Genomics Division, National Institute of Agricultural Sciences, RDA, Jeonju, 54874 Republic of Korea; 3 Department of Industrial Plant Science & Technology, Chungbuk National University, Cheongju, 28644 Republic of Korea; 4 Jeollabukdo ARES Medicinal Resource Research Institute, Jinan, 55440 Republic of Korea; 5 Department of Biological Sciences and Biotechnology, Chungbuk National University, Cheongju, 28644 Republic of Korea

**Keywords:** Fruit development, Lignan biosynthesis, Long-read transcriptome sequencing, Phenylpropanoid biosynthesis, *Schisandra chinensis*

## Abstract

**Background:**

*Schisandra chinensis*, an ancient member of the most basal angiosperm lineage which is known as the ANITA, is a fruit-bearing vine with the pharmacological effects of a multidrug system, such as antioxidant, anti-inflammatory, cardioprotective, neuroprotective, anti-osteoporosis effects. Its major bioactive compound is represented by lignans such as schisandrin. Molecular characterization of lignan biosynthesis in *S. chinensis* is of great importance for improving the production of this class of active compound. However, the biosynthetic mechanism of schisandrin remains largely unknown.

**Results:**

To understand the potential key catalytic steps and their regulation of schisandrin biosynthesis, we generated genome-wide transcriptome data from three different tissues of *S. chinensis* cultivar Cheongsoon, including leaf, root, and fruit, via long- and short-read sequencing technologies. A total of 132,856 assembled transcripts were generated with an average length of 1.9 kb and high assembly completeness. Overall, our data presented effective, accurate gene annotation in the prediction of functional pathways. In particular, the annotation revealed the abundance of transcripts related to phenylpropanoid biosynthesis. Remarkably, transcriptome profiling during fruit development of *S. chinensis* cultivar Cheongsoon revealed that the phenylpropanoid biosynthetic pathway, specific to coniferyl alcohol biosynthesis, showed a tendency to be upregulated at the postfruit development stage. Further the analysis also revealed that the pathway forms a transcriptional network with fruit ripening-related genes, especially the ABA signaling-related pathway. Finally, candidate unigenes homologous to isoeugenol synthase 1 (*IGS1*) and dirigent-like protein (*DIR*), which are subsequently activated by phenylpropanoid biosynthesis and thus catalyze key upstream steps in schisandrin biosynthesis, were identified. Their expression was increased at the postfruit development stage, suggesting that they may be involved in the regulation of schisandrin biosynthesis in *S. chinensis*.

**Conclusions:**

Our results provide new insights into the production and accumulation of schisandrin in *S. chinensis* berries and will be utilized as a valuable transcriptomic resource for improving the schisandrin content.

**Supplementary Information:**

The online version contains supplementary material available at 10.1186/s12864-021-08253-2.

## Background

*Schisandra chinensis*, also known as ‘Omija’ (Korea), ‘Wuweizi’ (China), or ‘Gomishi’ (Japan), is a climbing species with a generation time of five years that belongs to the order Austrobaileyales, which consists of early-diverging angiosperms [1]. The natural habitats of *S. chinensis* are mostly within northeastern Asia; they show a uniform distribution of genetic diversity and low genetic differentiation because of extensive gene flow [2]. The berry fruits of *S. chinensis* are clustered in grape-like bunches and exhibit five flavors: salty, sweet, sour, pungent (spicy), and bitter. The fruits contain many bioactive compounds, including lignans, triterpenes, phenolic acids, flavonoids, essential oils, and polysaccharides [3, 4], and they have been used as an important traditional medicine in northeastern Asia. In particular, dibenzocyclooctadiene lignans, including schisandrin and gomisin, present in *S. chinensis* fruit extracts exhibit pharmacological effects such as antitumor, anti-inflammatory, antioxidative, hepatoprotective, antihypertensive, and anti-osteoporosis activities [3–7]. Moreover, fruits have the potential to effectively protect against neuronal cell damage and to significantly enhance cognitive performance, suggesting their usefulness as new therapeutic agents for treating neurodegenerative diseases [8].

In general, biosynthesis of lignans in plants is linked to phenylpropanoid pathway in an upstream function and branches after synthesis of coniferyl alcohol. A succession of specific steps, involving in catalytic reactions of dirigent (DIR), pinoresinol-lariciresinol reductase (PLR), secoisolariciresinol dehydrogenase (SILD), *O*-methyltransferases (OMTs; i.e. OMT1 and OMT3), cytochrome P450 families (CYPs; i.e. CYP719A23, CYP71CU1, CYP71BE54, CYP82D61), and UDP-glucose-dependent glucosyltransferases (UGTs) [9–15], lead to production of lignans (Additional file 4: Fig. S7). The specific steps is closely related to the production of (−)-4′-desmethylepipodophyllotoxin, an etoposide aglycone, that was well characterized in *Podophyllum hexandrum* [14]. Interestingly, the genetic engineering of lignan biosynthetic enzyme genes (i.e. *PLR-RNAi* in *Forsythia*) demonstrated the direct alteration of the lignan production in plants [16]. Unlike podophyllotoxin biosynthetic pathway that starts with the synthesis of pinoresinol in lignan branch, dibenzocyclooctadiene lignan is initiated with the synthesis of isoeugenol [17]. In early steps for the biosynthesis of dibenzocyclooctadiene lignan, isoeugenol molecules are further metabolized to verrucosin and dihydroguaiaretic acid by catalytic reactions of DIR and PLR, respectively [17]. The resulting dihydroguaiaretic acid is converted to schisandrin or gomisin via several steps that are catalyzed by putative OMTs, CYPs and/or UGTs. However, the fully understanding of this biosynthetic pathway is still far from complete. Because schisandrin biosynthesis is distinct from podophyllotoxin biosynthetic pathway, it may be limited to understand schisandrin biosynthesis in *S. chinensis* based on podophyllotoxin biosynthetic pathway. Thus, prior to discovering key active enzymes related to schisandrin biosynthesis, the study of the regulation of genes, such as *DIR*, *PLR*, and *IGS1*, involved in early step of the biosynthetic pathway can provide insight into the production and accumulation of schisandrin.

The accumulation of polyphenolic compounds such as lignans is reported to be affected by fruit maturation stages, which are dependent on environmental conditions and the genetic makeup of a species [18, 19]. Fruits undergo various physiological and biochemical changes during maturation, consequently altering their bioactive composition. Although very little research has been conducted to investigate the changes in nutraceutical components during ripening in *S. chinensis*, the contents of lignans in the fruits of *S. chinensis* have been studied. Previous studies have reported high contents of schisandrin in the ripened fruits of *S. chinensis* [20, 21]. Szopa et al. [6] identified the highest contents of gomisin N, followed by schisandrin A, schisandrin, gomisin D, schisantherin B, gomisin A, angeloylgomisin H and gomisin J. In connection with the fruit ripening and the accumulation of polyphenols, the phenylpropanoid biosynthesis is known to activate their production and accumulation [22, 23], probably providing a clue that explains how schisandrin is synthesized by the activation of phenylpropanoid biosynthesis. Therefore, such information is useful for optimizing the harvesting time of a fruit species to achieve the maximum nutraceutical potential and provides insight for designing new strategies to improve the production of clinically effective components in *S. chinensis*.

Long-read transcriptome sequencing, such as isoform sequencing (Iso-Seq) via the single-molecule real-time (SMRT) sequencing, produces longer and more accurate transcripts with a high level of assembly completeness and gene annotation [24]. Thus, the sequencing approach has been recently utilized for functional genomics in large complex or uncharacterized plant species [24–26]. For example, Wang et al. [25] reported that maize yielded considerable nonredundant full-length transcript isoforms, corresponding to approximately 26,946 genes. In addition, the genome coverage of Iso-Seq data is near saturation with maximum transcriptome diversity. Thus, de novo transcriptome assembly using Iso-Seq will help in the exploration of genes related to the biosynthesis of major bioactive compounds in medicinal plants with no reference genome, and will facilitate understanding of the underlying mechanism that provides new insight to the physiological role of these metabolites and their functional diversity in plants. Ultimately, long-read transcriptome sequencing may be a cost-effective gold standard for building reference gene sets for conducting functional genomics in plants.

To obtain a more comprehensive view of the transcriptome in *S. chinensis*, especially insight into the regulation of lignan biosynthesis during fruit development, we generated de novo transcriptome data derived from three different tissues, including leaves, roots, and fruits, via long-read transcriptome sequencing coupled with RNA-Seq. Although the genomic information of *S. chinensis* is not yet sufficient, our data allowed highly accurate gene annotation with ORF prediction, thus being helpful for identifying candidate genes involved in lignan biosynthesis. Furthermore, we investigated the transcriptional changes of these genes during fruit development in *S. chinensis*. In particular, the analysis revealed a transcriptional network between phenylpropanoid biosynthesis and ripening-related phytohormones. Our data can be used as a valuable transcriptomic resource for discovering genes associated with the production of medicinal compounds in *S. chinensis* and are helpful for understanding the regulation of lignan biosynthesis in its fruit.

## Results

### De novo transcriptome assembly in *S. chinensis* via long- and short-read sequencing technologies

**Fig. 1:**
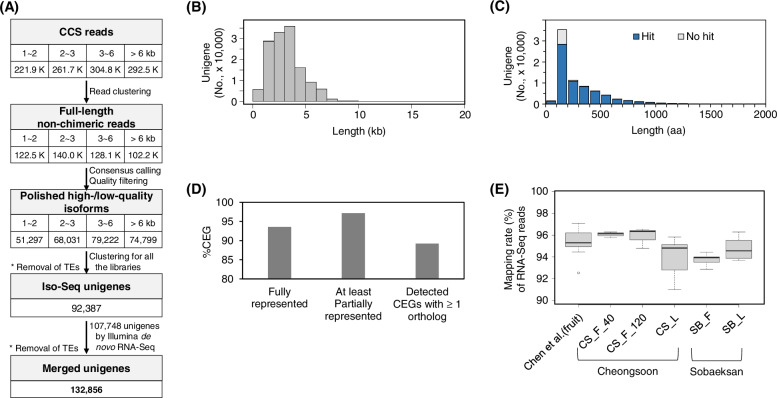
Overview of transcript assembly in *S. chinensis*. **a**. Procedure of transcript assembly for *S. chinensis*. Clean subreads of each size class (1 ~ 2 kb, 2 ~ 3 kb, 3 ~ 6 kb, and > 6 kb) were merged into the corresponding isoform cluster and classified as full-length (FL) and/or non-FL reads. After consensus sequence calling and quality filtration were carried out, the sequences were clustered into Iso-Seq unigenes. Unigenes derived from the Iso-Seq and Illumina de novo RNA-Seq procedures were merged after TE sequences were removed. **b**. The length distribution of unigenes. **c**. The length distribution of translated sequences (aa) predicted from unigenes. **d**. CEGMA evaluation. The completeness (%) of the unigenes (‘fully represented CEGs’) was assessed by CEGMA. **e**. Mapping of RNA-Seq samples derived from fruit and leaf tissues. In the figure, RNA-Seq data of four fruit, including Chen et al. [27] (from Jilin, China), CS_F_40 and CS_F_120 (sampled at 40 and 120 DAFs of fruit in Cheongsoon), and SB_F (Sobaeksan), and two leaf samples, including CS_L (Cheongsoon) and SB_L (Sobaeksan), were analyzed

Using PacBio Iso-Seq and llumina RNA-Seq, we generated de novo transcriptome assembly in a *S. chinensis* cultivar Cheongsoon that shows the characteristics of large fruits, high yielding ability, and tolerance to brown leaf spot or powdery mildew compared with a wild type Sobaeksan (Additional file 5; Table S4). We first generated large cDNA fragments from *S. chinensis* by using Iso-Seq (Fig. 1a), which favors the reverse transcription of intact, full-length mRNA molecules and identifies candidate genes related to interesting functional pathways, such as phenylpropanoid and lignan biosynthesis. cDNA libraries were size-selected for lengths of 1–2 kb, 2–3 kb, 3–6 kb, and > 6 kb from RNA samples of *S. chinensis* pooled from three tissues (leaves, roots, and fruits) and sequenced on the PacBio RS II platform. A total of 9.5 million subreads were generated (Additional file 1: Table S1) and merged into 51,297 to 79,222 isoform clusters (Additional file 3: Table S3). After consensus calling, quality filtration, and the removal of transposable element (TE) sequences, the sequences were clustered into a total of 92,378 assembled transcripts, referred to as unigenes. Furthermore, to cover small transcripts, especially those of less than approximately 1 kb, which may be missed by size selection in Iso-Seq library construction, a total of 107,748 unigenes (N50 of 429 bp) that were de novo assembled using Illumina RNA-Seq data derived from fruit tissue were merged into Iso-Seq unigenes. A total of 132,856 unigenes were finally obtained with a cumulative length of approximately 246.9 Mb. This procedure is summarized in Fig. 1a. The average length of the unigenes was 1.9 kb, with a distribution of 2 ~ 4 kb (Fig. 1b); this average length was longer than that previously reported by Chen et al. [27] of 754 bp. Open reading frames (ORFs) were predicted in 73,774 unigenes (55.5%), accounting for a cumulative length of approximately 63.9 Mb, with an average length of 288 aa (Fig. 1c). Remarkably, 56,038 of these unigenes were predicted to contain complete full-length ORFs, most of which were generated from Iso-Seq (Additional file 4: Fig. S1).

We assessed the quality of the *S. chinensis* unigenes. First, the Core Eukaryotic Genes Mapping Approach (CEGMA), which assesses a highly reliable set of gene annotations in genome and transcriptome assemblies, revealed a completeness of 93.6% (Fig. 1d). Second, various RNA-Seq samples that were generated from leaves and fruits of Cheongsoon and Sobaeksan were mapped to the unigene dataset with coverage ranging from 91.0 to 97.1% (Fig. 1e), showing high data mapping coverage. Therefore, our data indicate the high quality of the assembly and mapping coverage of the reads for *S. chinensis*, which is a helpful transcriptome reference dataset.

### Functional annotation of unigenes

**Fig. 2:**
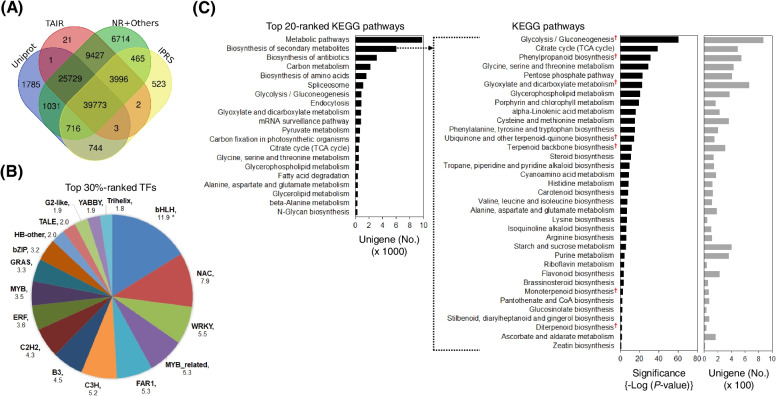
Overview of the functional annotation of unigenes in *S. chinensis*. **a**. Overlap of hits among known homologous genes obtained from searches against different databases, including UniProt, TAIR, NR, and InterPro. **b**. The primary TF families identified in unigenes by PlantTFDB search. **c**. Abundance of unigenes assigned to the top 20 ranked KEGG pathways (left panel) and enrichment of unigenes related to secondary metabolite biosynthesis in the *S. chinensis* transcriptome dataset (right panel) [28, 29]. KEGG enrichment analysis was performed by using DAVID according to an EASE score < 0.001. Cross mark (‘†’) indicates the primary KEGG pathway focused on in this study

All unigenes were annotated using homology-based searches. Of all unigenes, 90,930 unigenes (68.4%) showed significant homology with protein sequences in the UniProt (69,782 hits), TAIR (78,952 hits), NCBI NR (87,851 hits), and InterPro (46,222 hits) databases. In the searches, 83,228 (91.5%) annotated unigenes were predicted to be known genes based on UniProt and TAIR, which accept primary sequences derived from sequencing experiments with functional information, showing good annotation quality. In particular, 39,776 of the known unigenes were predicted to be known genes based on the identification of protein domains (Fig. 2a), showing outstanding hit scores. Of the annotated unigenes, 75,748 were identified to be derived from Iso-Seq data, showing efficient transcript annotation by long-read sequencing. We also examined unigenes homologous to known transcription factor (TF) sequences by searching against PlantTFDB using BLASTX with a cutoff of 1*E*-05. A total of 21,800 unigenes classified into 58 TF families were identified. In the search, the bHLH, NAC, WRKY, MYB/−related, FAR1, C3H, B3, C2H2, ERF, GRAS, bZIP, HB-other, TALE, G2-like, YABBY, and Trihelix families were abundant in *S. chinensis*, making up the top 30% of all families according to abundance (Fig. 2b). Interestingly, bHLH, NAC, MYB, ERF, GRAS, bZIP, and TALE are involved in fruit development and/or plant growth [30]. The predominant proteins in terms of Pfam domains included P-loop-containing nucleotide triphosphate hydrolase (5.4%), various protein kinases (17.1%), tetra- and penta-tricopeptides (5.7%), and NAD(P)-binding protein (2.1%) (Additional file 4: Fig. S2a). In addition, UniProtKB keywords associated with functional membranes (7%), alternative splicing (3.7%), transferases (3.7%), the nucleus (3.6%), and binding (6.2%) were highly abundant (Additional file 4: Fig. S2b).

To further predict and classify unigene functions, the annotated unigenes were analyzed according to KEGG pathway assignments. A total of 12,940 unigenes were assigned to 20 primary pathways. The most common pathways were ‘metabolic pathways’, ‘biosynthesis of secondary metabolites’, ‘biosynthesis of antibiotics’, ‘carbon metabolism’, ‘terpenoid biosynthesis’, and ‘biosynthesis of amino acids’ (left panel in Fig. 2c). In particular, the enrichment analysis for transcripts related to ‘biosynthesis of secondary metabolites’ revealed the significance of pathways related to ‘glycolysis/gluconeogenesis’, ‘TCA cycle’, ‘glyoxylate and dicarboxylate metabolism’, ‘phenylpropanoid biosynthesis’, ‘amino acid-related metabolism’, ‘lipid-related metabolism’, ‘terpenoid biosynthesis’, and ‘carotenoid biosynthesis’ (right panel in Fig. 2c). Interestingly, a high abundance of transcripts related to phenylpropanoid biosynthesis was identified. This result seems to suggest that the activation of the phenylpropanoid pathway in *S. chinensis* is associated with the biosynthesis of polyphenols such as lignans or flavonoids [22, 23]. Our data show that long-read-based transcriptome sequencing resulted in more effective, accurate annotation or prediction for elucidating genes associated with interesting traits or functional pathways in *S. chinensis*.

### Characterization of transcripts in phenylpropanoid and lignan biosynthesis

**Fig. 3:**
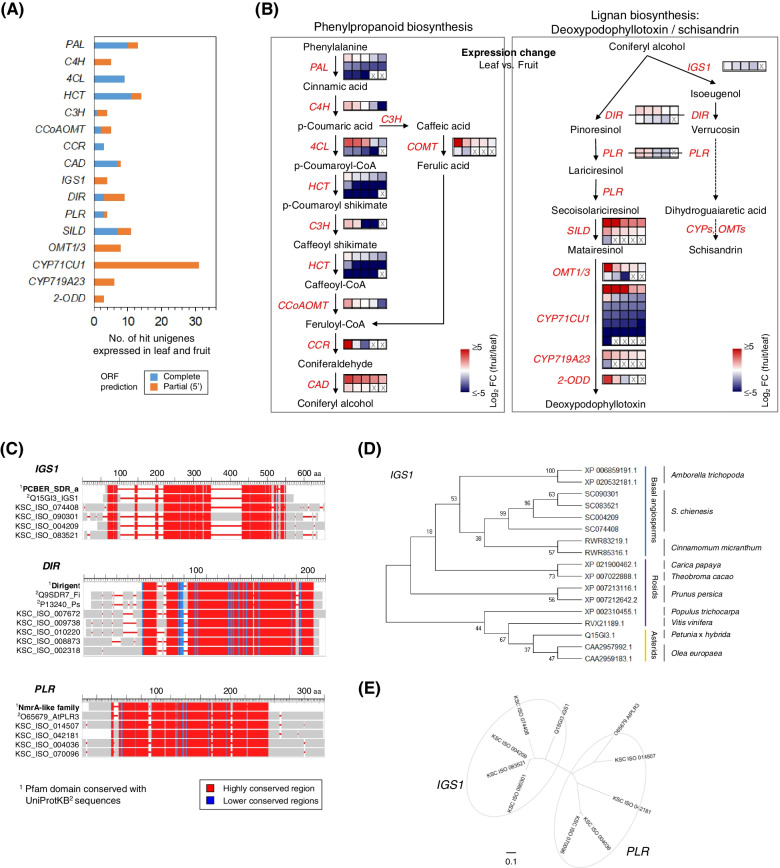
Identification of candidate genes related to phenylpropanoid and lignan biosynthetic pathways in *S. chinensis*. **a**. Identification of unigenes homologous to genes related to these two biosynthetic pathways. **b**. Expression of candidate unigenes in the two pathways in leaf and fruit tissues. After quantifying gene expression from RNA-Seq data derived from the leaves and fruits of *S. chinensis* (Cheongsoon) with five replicates, the log_2_-scale fold changes of each of gene between leaf and fruit were calculated. In Figure, each small square cell indicates unigene homologous to gene encoding enzyme involved in the pathways. In addition, expression change of each unigene between leaf and fruit is shown with heatmap. Mark ‘x’ in small square cell indicates no unigene. **c**. Multiple-sequence alignment of *DIR*, *IGS1*, and *PLR* homologs. Sequence conservation was identified among targeted genes (UniProt accession number: Q9SDR7 for *DIR*, Q15G13 for *IGS1*, and O65679 for *PLR*), the corresponding primary protein domains (dirigent for *DIR*, PCBER_SDR_a for *IGS1*, and NmrA-like family for *PLR*), and the corresponding homologous unigenes. **d.** Phylogeny of *IGS1* homologs on the basis of the relationships among major linages of angiosperms sequenced, including basal angiosperms (GenBank accession number: XP_006859191.1 and XP_020532181.1 *Amborella trichopoda* and RWR83219.1 and RWR85316.1 for *Cinnamomum micranthum), rosids (XP_021900462.1 for Carica papaya, XP_007022888.1 for Theobroma cacao*, XP_007213116.1 and XP_007212642.2 for *Prunus persica, XP_002310455.1 for Populus trichocarpa, and RVX21189.1 for Vitis vinifera), asterids (Q15GI3.1 for Petunia x hybryda and CAA2957992.1 and CAA2959183.1 for Olea europaea)* reported by the Amborella Genome Project [32]. **e**. Phylogenetic analysis between *IGS1* and *PLR* homologs via the maximum likelihood-based method

In relation to the lignan biosynthetic pathway, in the podophyllotoxin pathway, especially the pathway leading to etoposide aglycone, (−)-4′-desmethylepi podophyllotoxin, an identified biosynthetic gene, has been studied in detail [14]. However, the biosynthetic mechanism of schisandrin production remains largely unknown, and only early steps for catalyzing the synthesis of dihydroguaiaretic acid that is converted to the dibenzocyclooctadiene skeleton have been studied [31]. Nevertheless, to understand lignan biosynthesis in *S. chinensis*, we identified transcripts related to lignan biosynthetic pathways for deoxypodophyllotoxin and dihydroguaiaretic acid and the phenylpropanoid biosynthetic pathway for coniferyl alcohol, which is utilized for activating the lignan biosynthetic pathway (Fig. 3b). For phenylpropanoid biosynthesis, a total of 61 homologous unigenes were identified: 13 *PALs*, 5 *C4Hs*, 9 *4CLs*, 14 *HCTs*, 4 *C3Hs*, 5 *CCoAOMTs*, 3 *CCRs*, and 8 *CADs* (Fig. 3a). For lignan biosynthesis, 76 homologous unigenes were also identified: 4 *IGSs*, 9 *DIRs*, 4 *PLRs*, 11 *SILDs*, 8 *OMT1/3 s-like*, 31 *CYP71CU1s-like*, 6 *CYP719A23/24 s-like*, and 3 2-*ODDs* (Fig. 3a and b). This result shows the conservation of those biosynthetic pathways in *S. chinensis*. Of those unigenes identified, unigenes homologous to *PAL*, *HCT*, *4CL* and *CAD* for phenylpropanoid biosynthesis and *DIR*, *SILD*, *OMT1/3*, and *CYP71CU1* for lignan biosynthesis were relatively abundant (Fig. 3a and b).

Among the identified unigenes, full-length ORFs homologous to *IGS, DIR* and *PLR,* which essentially contribute to the early step in the biosynthesis of the two lignans, were identified by analyzing conserved protein domains through multiple-sequence alignment with previously reported target genes (Fig. 3c). First, four *IGS1* unigenes, KSC_ISO-074408, − 090301, − 004209 and − 08352, which were distinct from eugenol synthase 1 (*EGS1*) or isoflavone reductase, were identified to contain a phenylcoumaran benzylic ether reductase (PCBER)-like protein domain. *IGS1* is an atypical short-chain dehydrogenase/reductase (SDR) identified in *Petunia hybrid* [33] that acts as an NADPH-dependent aromatic alcohol reductase and functions to catalyze the synthesis of phenylpropene isoeugenol from coniferyl alcohol. *IGS1* homologs identified in *S. chinensis* were subordinate to the basal angiosperm group (Fig. 3d) compared with other key lineages that correspond to mostly tree genomes. Second, five *DIR* unigenes, including KSC_ISO-007672, − 009738, − 010220, − 008873 and − 002318, were identified with high sequence conservation to the dirigent protein domains of *psd_Fi1* and *PI206* identified in *Forsythia intermedia* [34] and *Pisum sativum* [35]. *DIR* plays a role in selectively biosynthesizing pinoresinol or verrucosin from two molecules of coniferyl alcohol. Sequence divergences of those *DIR* unigenes in *S. chinensis* were identified in their 5′ and 3′ regions. Third, four *PLR* unigenes, including KSC_ISO-014507, − 042181, − 004036 and − 070096, were identified with high sequence conservation to *AtPLR3* of *Arabidopsis*, which contains a *NmrA*-type oxidoreductase family protein domain and might be involved in the reduction of lariciresinol into secoisolariciresinol [10, 36]. Interestingly, although *IGS1* and *PLR* harbored NAD(P)-binding motifs (as members of the SDR family), they showed clear sequence divergence (Fig. 3e). These identified genes are potential candidates for elucidating the regulation of lignan biosynthesis in *S. chinensis*.

### Differential expression of transcripts involved in phenylpropanoid and lignan biosynthesis in the fruit and leaf of *S. chinensis*

We also examined the differential expression patterns of transcripts involved in phenylpropanoid and lignan biosynthesis by analyzing RNA-Seq data derived from the deep red-colored fruit and yellow green leaf of *S. chinensis* cultivar Cheongsoon at 120 days after flowering (DAF) that may lead to the two biosynthesis at the high level. Overall, most unigenes showed divergent expression in the two tissues (Fig. 3b). Transcriptome diversity was even identified among paralogs or isoforms. These results suggest that the tissue-specific expression of transcripts is involved in these two biosynthetic pathways. In phenylpropanoid biosynthesis, most *PAL* and *HCT* homologs showed higher expression patterns in the leaves, but all *CAD* homologs were remarkably upregulated in the fruit (left panel of Fig. 3b), suggesting the predominant production of coniferyl alcohol by CAD in the fruit *S. chinensis*. Although indirect evidence was obtained, the high content of schisandrin in the fruit of *S. chinensis* was actually identified by high-performance liquid chromatography (HPLC) profiling (significance at *P* < 0.01) (Additional file 4: Fig. S3), supporting this hypothesis. In those two pathways for deoxypodophyllotoxin and dihydroguaiaretic acid, three *IGS1* unigenes showed slightly high expression in the leaf, but three *DIR* and two *PLR* unigenes showed selectively high expression in the fruit with small fold-changes (right panel of Fig. 3b; KSC_ISO-009738 (1.7-fold change) and − 010220 (2.0-fold change) for *DIR*, and KSC_ISO-014507 (1.8-fold change) and − 042181 (2.2-fold change) for *PLR*). Interestingly, most *SILD* unigenes, which are involved in matairesinol biosynthesis, were upregulated in the fruit, especially KSC_ISO_081641 (71.4-fold change in the fruit) (right panel of Fig. 3b), suggesting the predominant production of matairesinol in the fruit of *S. chinensis*. Our results show the tissue-specific expression of transcripts in the phenylpropanoid and lignan biosynthetic pathways in *S. chinensis* and their expression diversity.

### Transcriptome profiling during fruit development in *S. chinensis*

**Fig. 4:**
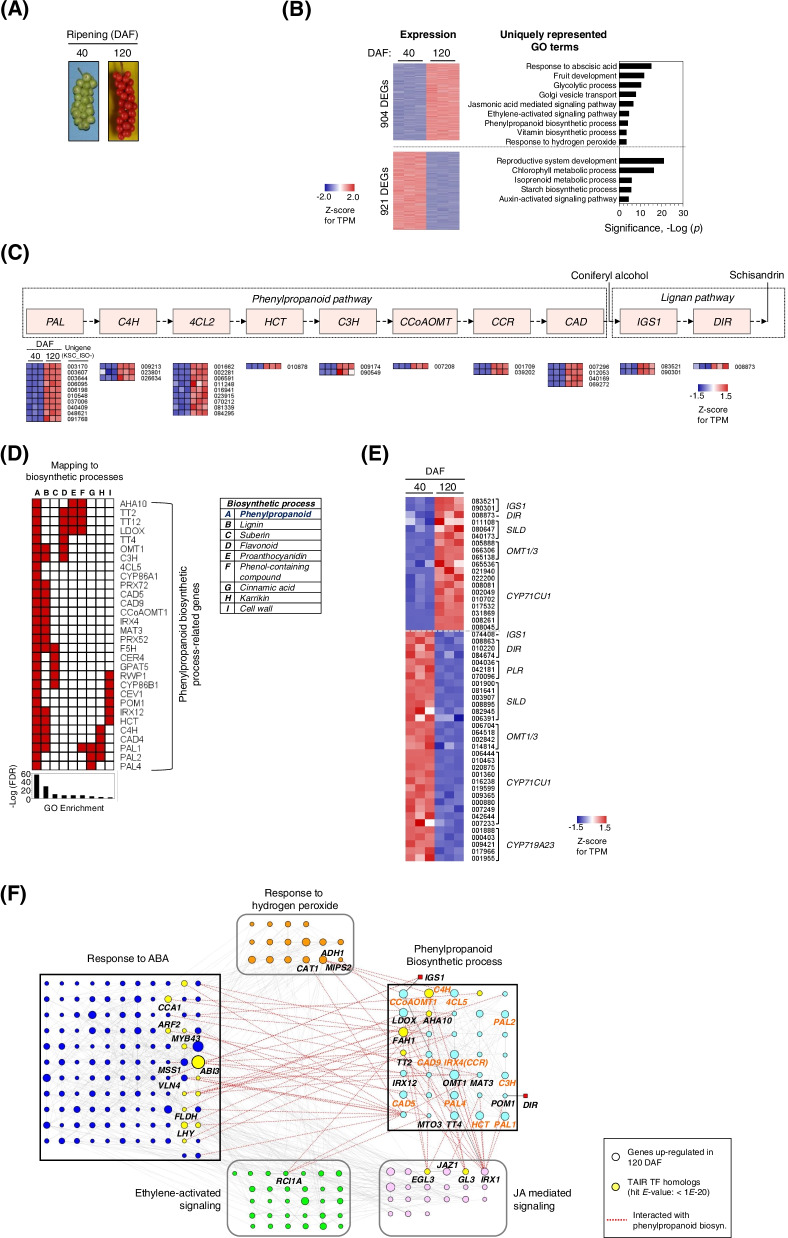
Profile of differentially expressed genes (DEGs) between the green- and red-colored berry stages of *S. chinensis*. **a**. Fruit shape at 40 (green-colored berry) and 120 (red-colored fruit berry) days after flowering (DAF) in *S. chinensis*. **b**. Gene ontology (GO) enrichment for DEGs between 40 DAF and 120 DAF. The GO enrichment analysis for up- (904 genes) and down- (921 genes) regulated genes at 120 DAF (left panel in Figure) was performed by using DAVID according to an EASE score < 0.001, and significant, uniquely represented GO terms were then selected. **c**. Activation of the phenylpropanoid biosynthetic pathway to coniferyl alcohol at 120 DAF and identification of the corresponding unigenes. **d**. Mapping to genes in the phenylpropanoid biosynthetic process to other closely related biosynthetic processes, including lignin, suberin, flavonoid, proanthocyanidin, phenol-containing compound, cinnamic acid, karrikin, and cell wall biosynthetic processes. **e**. Identification of DEGs related to lignan biosynthetic pathways at 40 and 120 DAF. **f**. Transcriptional network among genes upregulated at 120 DAF related to phenylpropanoid biosynthesis and the responses to ABA, JA, ethylene, and hydrogen peroxide. After selecting up-regulated genes enriched in those five GO terms, the interaction between those genes was identified by using STRING and further analyzed using Cytoscape on the basis of the degree of connectivity of the nodes. In the network, the interaction between genes in ‘phenylpropanoid biosynthetic process’ and other functional terms, including ‘responses to ABA’, ‘JA mediated signaling’, ‘ethylene-activated signaling’, or ‘response to hydrogen peroxide’, are indicated with red-color dot line. In addition, genes in phenylpropanoid pathway are indicated with orange-color letter

The amounts of dibenzocyclooctadiene lignans in the fruits of *S. chinensis* are known to be influenced by the degree of fruit maturity and harvest time [3]. To understand transcriptomic changes during fruit development in *S. chinensis*, we analyzed differentially expressed genes (DEGs) using RNA-Seq data derived from two different fruit development stages in *S. chinensis* cultivar Cheongsoon, 40 (green-colored and circle-shaped fruit berry) and 120 (deep red-colored and circle-shaped fruit berry) DAF (Fig. 4a), with cutoffs of *q* < 0.05, an absolute fold change ≥1.5, and an TPM ≥ 1 as the minimum average expression value between genes in the two stages. A total of 16,698 DEGs, consisting of 9344 downregulated and 7354 upregulated genes at 120 DAF, were identified and subjected to functional categories with Gene Ontology (GO) enrichment analysis. The GO enrichment analysis for genes upregulated at 120 DAF, 904 DEGs, revealed the significance of the functions associated with ‘fruit development’ (1.39 × 10^− 12^), ‘Golgi vesicle transport’ (1.04 × 10^− 8^), ‘response to plant hormone signaling pathways’ such as abscisic acid (ABA) (4.95 × 10^− 16^), ethylene (1.83 × 10^− 5^), and jasmonic acid (JA) (1.6 × 10^− 7^), ‘phenylpropanoid biosynthetic process’ (6.87 × 10^− 5^), ‘vitamin biosynthetic process’ (2.69 × 10^− 4^), and ‘response to hydrogen peroxide (H_2_O_2_)’ (3.34 × 10^− 4^) (Fig. 4b). Remarkably, the pathway for coniferyl alcohol production within the phenylpropanoid biosynthetic pathway is likely to be activated as a specific synthesis route of lignan. In this pathway, the expression of 10 *PAL*, 3 *C4H*, 9 *4CL2*, 1 *HCT*, 2 *C3H*, 1 *CCoAOMT*, 2 *CCR* and 4 *CAD* unigenes was significantly increased (Fig. 4c). In particular, three *CAD* homologous unigenes (KSC_ISO-012053, − 040169 and − 069272) showed an increase in expression with ≥2.7 ~ 10-fold changes (Additional file 4: Fig. S4). As presented in Additional file 4: Fig. S6, the qRT-PCR data for these DEGs were generally similar with the RNA-seq data, showing a positive correlation (*r* = 0.601). Interestingly, the phenylpropanoid biosynthetic pathway was extensively linked to biosynthetic processes such as those for lignin (FDR = 5.33 × 10^− 30^), suberin (3.54 × 10^− 11^), proanthocyanidin (1.04 × 10^− 8^), flavonoid (8.04 × 10^− 9^), phenol-containing compound (2.09 × 10^− 8^), cinnamic acid (2.53 × 10^− 6^), karrikin (1 × 10^− 4^), and cell wall (2.2 × 10^− 3^) (Fig. 4d). This result supports the potential for the activation of other secondary metabolites by the genes involved in the phenylpropanoid biosynthetic pathway at the postfruit development stage of *S. chinensis*. Therefore, our results indicate the activation of the phenylpropanoid biosynthetic process for lignan biosynthesis at the postfruit development stage of *S. chinensis*. Moreover, genes related to the response to ABA, ethylene-activated signaling, and response to H_2_O_2_, which are known to be associated with fruit ripening [37], are upregulated, implicating a functional link between lignan biosynthesis and ripening. We also identified unigenes upregulated in lignan biosynthetic pathways at 120 DAF (Fig. 4e): 2 *IGS1s*, 1 *DIR,* 3 *SILDs,* 3 *OMT1/3*, and 10 *CYP71CU1*. However, unlike *IGS1* and *DIR* unigenes, *PLR* unigenes showed upregulation at 40 DAF. Additionally, most *SILD* and *CYP719A23* unigenes were upregulated at 40 DAF. Collectively, our results show the transcriptional changes in the phenylpropanoid and lignan biosynthetic pathways at postfruit development in *S. chinensis*.

### Transcriptional network between phenylpropanoid biosynthesis and plant hormone signaling

To investigate the functional links between phenylpropanoid biosynthesis and ripening-related plant hormone signaling in *S. chinensis*, a transcriptional network of genes upregulated at 120 DAF, which were enriched in the functional categories of ‘phenylpropanoid biosynthetic process’ and ‘responses to plant hormone signaling pathways’, including ABA, ethylene, and JA, and ‘response to H_2_O_2_’, was analyzed. The analysis showed that the phenylpropanoid biosynthetic pathway highly interacted with the ABA and JA signaling pathways (Fig. 4f). In the network, *CYP84A1* (*FAH1*), *LDOX*, *CAD5*, *TT2*, *IRX12*, *AHA10*, and *OMT1*, which are involved in the phenylpropanoid biosynthetic process, interacted with *ABI3*, *LHY*, *CCA1*, *MSS1*, *FLDH*, *MYB43*, and *ARF2* during ABA signaling and *IRX1*, *GL3*, and *EGL3* during JA signaling. Three genes, *AHA10*, *MTO3*, and *MAT3,* for the phenylpropanoid biosynthetic process, also interacted with *RCI1A* during ethylene signaling, which is linked to ABA signaling. In connection with plant hormone signaling, *IGS1* and *DIR* interacted with *CCoAOMT1* and *POM1* in the phenylpropanoid biosynthetic process, respectively, and *IGS1* was linked to *VLN4*. Additionally, the hydrogen peroxide (H_2_O_2_) signaling pathway, including *CAT1*, *ADH1* and *MIPS2*, also interacted with genes involved in phenylpropanoid biosynthetic processes, including *TT4, CCoAOMT1, C4H,* and *4CL5*. Hydrogen peroxide signaling play an important role in promoting ripening at the molecular level [38]. However, the functions of those three genes associated with fruit ripening remain unknown. These results indicate that the phenylpropanoid biosynthetic pathway is closely connected with the ABA, JA, ethylene, and hydrogen peroxide signaling pathways in the postfruit development stage of *S. chinensis* and that crosstalk may affect the activation of lignan biosynthesis.

## Discussion

### Generation of high-quality reference transcriptome data for *S. chinensis*

We generated the de novo transcriptome assembly of *S. chinensis* cultivar Cheongsoon to obtain insights into the biosynthesis of schisandrin. Because Cheongsoon has the characteristics of high yielding ability and disease tolerance compared with wild type, it has been considered as a plant material for constructing a reference transcriptome in *S. chinensis*. In addition to the de novo transcriptome assembly of Cheongsoon, we have been working on the project for whole-genome de novo assembly of Cheongsoon. To generate longer unigenes and cover small transcripts in Cheongsoon, a hybrid assembly using long- and short-read sequencing data was employed. This approach showed that the resulting unigene dataset presented a high level of transcript assembly completeness and deep data coverage of the diversity of the transcriptome. Thus, this dataset can lead to the accurate quantification of gene expression and the identification of interesting or novel transcripts of large genes in the absence of whole-genome sequence information [39]. Remarkably, our transcriptome data presented considerable unigenes containing complete ORFs with full-length transcripts. This result indicates the potential for effective, accurate gene annotation for elucidating genes associated with traits or functional pathways of interest. For example, a total of 83 full-length unigenes related to phenylpropanoid and lignan biosynthesis in *S. chinensis* could be identified with high homology scores of <1*E*-20 (Fig. 3), and the conservation of pathways to coniferyl alcohol, deoxypodophyllotoxin, and schisandrin biosynthesis in *S. chinensis* was identified. In particular, the successful identification of *IGS1*, *DIR*, and *PLR* will be helpful for genome engineering to improve traits associated with pharmacological effects in the fruit of *S. chinensis*. For such reasons, a long-read sequencing-based approach has been recently favored in plants such as red clover, *Arabidopsis pumila*, grape berry, *Taxus cuspidate,* and *Gossypium austral* [40–43], which have not yet undergone whole-genome sequencing. Therefore, our transcriptome data are very useful as reference data for functional genomics and genomic resources for gene prediction and molecular marker development in *S. chinensis*.

### Abundance of transcripts related to phenylpropanoid biosynthesis, terpenoid biosynthesis, and gluconeogenesis in *S. chinensis* transcriptome data

The functional annotation of the *S. chinensis* transcriptome data revealed the relatively high abundance of transcripts related to phenylpropanoid biosynthesis, terpenoid biosynthesis, and gluconeogenesis. This finding is likely to indicate the predominant activation of those biosynthetic pathways in *S. chinensis*. In fruit crops, the phenylpropanoid biosynthetic pathway is known to activate the production and accumulation of polyphenols such as lignans and flavonoids [22, 23, 44, 45]. With previous reports, our results provide a clue that explains how lignans such as shisandrins are essentially synthesized by the phenylpropanoid biosynthetic pathway in *S. chinensis*. Interestingly, transcripts related to the pathway exhibited divergent expression in leaves and fruits, implicating functional diversity between different tissues or among isoforms or paralogs. In addition to phenylpropanoid biosynthesis, the abundance of transcripts related to terpenoid biosynthesis was also identified. This result suggests that terpenoids (i.e., wuweizidilactones) in *S. chinensis* might be one of the primary compounds, with lignans having dibenzocyclooctadiene skeletons. The biosynthesis of terpenoids in *S. chinensis* is not yet known. Some recent studies have reported the identification of new triterpenoids and their bioactivity with weak or no cytotoxicity [46]. We also identified the highest abundance of transcripts related to gluconeogenesis in the transcriptome dataset of *S. chinensis* (Additional file 4: Fig. S5). Interestingly, gluconeogenesis and the phenylpropanoid biosynthetic pathway are closely linked to fruit ripening processes that lead to the accumulation of sugar and subsequent changes in fruit tissue texture, as well as those that affect phenolic compounds [22, 47]. Moreover, the activation of the glyoxylate cycle, especially the upregulation of malate synthases, during fruit ripening plays an important role in gluconeogenesis via the conversion of lipids to carbohydrates [48–50].

### Activation of the phenylpropanoid biosynthetic pathway for lignan biosynthesis at the postfruit development stage of *S. chinensis* and its transcriptional network related to the ABA signaling pathway

In this study, we identified that the phenylpropanoid biosynthetic pathway tends to be activated for lignan biosynthesis at the postfruit development stage in *S. chinensis*. This finding was demonstrated by the GO enrichment analysis for genes upregulated in the 120 DAF sample (Fig. 4b). In particular, the analysis revealed that many of genes in the pathway leading to coniferyl alcohol, including *PAL*, *C4H*, *4CL2*, *HCT*, *C3H*, *CCoAOMT*, *CCR* and *CAD*, are up-regulated (Fig. 4c). Furthermore, the activation of the pathway is also connected to the upregulation of *IGS1* and *DIR*, which regulate the early steps of schisandrin biosynthesis (Fig. 4c). This result is similar with the finding by Zhang et al. [51]. Koeduka et al. [33] demonstrated that the overexpression of *PhIGS1* from petunia induced the accumulation of isoeugenol. Therefore, the up-regulation of genes related to phenylpropanoid biosynthetic pathway at the postfruit development stage seems to elicit the increase of production and accumulation of schisandrin in *S. chinensis*. In addition to the phenylpropanoid biosynthetic pathway, functions related to the response to ABA, ethylene-activated signaling, and response to H_2_O_2_ were also enriched, with upregulation at the postfruit development stage. These functions that are associated with fruit ripening are closely linked to the activation of the phenylpropanoid biosynthetic pathway and the consequent production and accumulation of lignans [22, 37]. This result is also supported by our findings that the activated phenylpropanoid biosynthetic pathway highly interacts with fruit ripening-related genes, especially those in the plant hormone signaling pathways of ABA, ethylene, and JA. Interestingly, of those three plant hormone signaling pathways, ABA is likely to be highly associated with lignan biosynthesis. ABA plays a key role in the regulation of *PLR* expression, which encodes a pinoresinol lariciresinol reductase, and lignan accumulation in *Linum usitatissimum*, which requires two *cis*-acting elements, *ABA* response element (*ABRE*) and *MYB2*, for this regulation [52]. The motif matrices of transcription factor ABI3 has significant affinity for the *DIR3* promoter with seed-specific expression [53]. MYB transcription factors *MYB43, MYB20,* and *MYB85* are transcriptional regulators that directly activate lignin and phenylalanine biosynthesis genes during secondary wall formation in *Arabidopsis* [54]. Additionally, in relation to JA, the bHLH transcription factor *EGL3* and its close homolog *GL3* are important regulators of the anthocyanin pathway in *Arabidopsis thaliana* [55]. Collectively, our results suggest that the activation of phenylpropanoid biosynthesis at the postfruit development stage is involved in the regulation of *IGS1* in lignan biosynthetic pathway. Moreover, genes related to ABA hormone signaling, especially ABI and MYB transcription factors, that is associated with fruit ripening and highly interact with phenylpropanoid biosynthetic pathway are also likely to be involved in the regulation of *DIR* and *PLR* in lignan pathway, thus contributing to the production and accumulation of schisandrin.

## Conclusions

We generated a longer, high-quality unigene dataset to obtain a more comprehensive view of lignan biosynthesis in *S. chinensis*. These data can be effectively utilized as reference data for the functional genomics of *S. chinensis*. Our transcriptome data cover transcripts for elucidating lignan and its precursor biosynthetic pathways. Remarkably, this study suggests that the activation of the phenylpropanoid biosynthetic pathway, along with the formation of a transcriptional network with fruit ripening-related genes, at the postfruit development stage of *S. chinensis* is highly linked to the production and accumulation of schisandrin. Therefore, our results will provide insight into lignan biosynthesis during fruit development in *S. chinensis*.

## Materials and methods

### Plant sampling and RNA preparation

Three tissues, leaves, roots, and fruits, from the *S. chinensis* cultivar Cheongsoon were collected (Additional file 1: Table S1; Additional file 2: Table S2). The cultivar Cheongsoon and a wild type Sobaeksan has been grown in the natural environment at the Medicinal Herb Resource Research Institute at Jeonbuk Agricultural Research & Extension Services (JABARES) in Jinan, Republic of Korea. The voucher specimens for Cheongsoon (MJ3–7) and Sobaeksan (SB-1) were deposited at the herbarium of the Medicinal Herb Resource Research Institute at JABARES. All of the tissues were cut into small pieces and frozen in liquid nitrogen, and ≥ 1 μg of total RNA from each sample was then extracted using an easy spin RNA extraction kit (iNtRON Biotechnology) according to the manufacturer’s instructions. The quality of the total RNA was assessed on an Agilent 2100 Bioanalyzer (Agilent Technologies, Santa Clara, CA) according to the criteria of an RNA integrity number (RIN) of 7.1 and a 28S/18S ratio of 1.0 on average.

### Iso-Seq and RNA-Seq

For PacBio Iso-Seq, one microgram of an RNA mixture obtained from equal amounts of RNA from the three tissues of Cheongsoon was reverse transcribed using the Clontech SMARTer cDNA synthesis kit (Clontech Laboratories, CA, USA). cDNA synthesis was followed by size selection according to length categories of 1–2 kb, 2–3 kb, 3–6 kb, and > 6 kb using BluePippin (Sage Science, Beverly, MA). The products were purified using AMPure PB beads (Pacific Biosciences, USA). The amplified and size-selected cDNA products were employed to generate SMRTbell template libraries according to the PacBio Iso-Seq protocol (Pacific Biosciences, Menlo Park, CA, USA). The libraries were prepared for sequencing via the annealing of a sequencing primer (component of the SMRTbell Template Prep Kit 1.0), followed by polymerase binding to the primer-annealed template. SMRT cells were sequenced on the PacBio RS II platform (Pacific Biosciences, Menlo Park, CA, USA) using P6-C4 chemistry with 3 ~ 4 h movies (Additional file 1: Table S1).

For Illumina RNA-Seq analysis, RNA-Seq libraries were prepared from 1 μg of total RNA derived from fruit and leaf using a TruSeq RNA Sample Prep Kit according to the manufacturer’s manual (Illumina, Inc., San Diego, CA). cDNA was synthesized from the mRNA fragments and was then subjected to an end repair process, the addition of a single ‘A’ base, and the ligation of the adapters. The libraries were purified and enriched via PCR amplification and were then subjected to paired-end sequencing with a 100 bp read length on the Illumina HiSeq 4000 platform (Additional file 2: Table S2).

### De novo transcript assembly

Iso-Seq raw reads were processed with PacBio SMRTlink (version 8.0) (https://www.pacb.com/support/software-downloads). First, reads of inserts (ROIs) were generated according to a minimum read quality of 75. ROIs were classified into full-length nonchimeric and non-full-length reads by identifying the 5′ and 3′ adapters employed in library preparation using Classify. Full-length reads were defined as those containing both adapters. All of the full-length reads were clustered using Cluster, and the clustered consensus sequences were then polished together with the non-full-length reads using Quiver. The high- and low-quality consensus sequences were clustered once more using CD-HIT (version 4.6.6) [56] with a sequence identity threshold of 0.99. De novo assembly using RNA-Seq was conducted with Trinity (version 2.0.4) [57]. After assembly, nonredundant representative sequences (referred to as unigenes) were generated by using the TGI Clustering Tool (TGICL) [58] and CD-HIT (version 4.6.6) [56]. After de novo assembly, unigenes belonging to TE sequences that were screened using RepeatMasker (version 4.0.6) (http://repeatmasker.org) were removed. The unigenes resulting from the Iso-Seq and RNA-Seq assemblies were merged. In the final unigene dataset, protein coding sequences (CDSs) were analyzed by using TransDecoder (version 3.0.0) [59] with the following steps: (1) a search for all possible CDSs, (2) verification of CDSs using GeneID [60], and (3) selection of the region with the highest score. To assess the quality of the assembled transcripts (or unigenes), CEGMA (version 2.5) [61], which assesses a highly reliable set of gene annotations during genome and transcriptome assembly, was employed together with the Eukaryotic Orthologous Groups of proteins (KOG) database. Moreover, the compatibility of unigenes with other datasets was analyzed by mapping with BWA (version 0.7.10) [62] using RNA-Seq samples derived from leaf and fruit tissues with the following parameters: -k 19 for the minimum seed length, −A 1 for the matching score, −B 4 for the mismatch penalty, and -T 30 for the alignment output with a score higher than 30.

### Functional annotation of unigenes

Unigenes were searched against the UniProt, NCBI nonredundant (NR), TAIR, and PlantTFDB databases using BLASTX (version 2.2.29+) [63] with a cutoff *E*-value of 1*E*-5. Protein domains were also searched using InterProScan (version 5.17–56.0) [64]. GO and Kyoto Encyclopedia of Genes and Genomes (KEGG) pathway annotations were performed using Blast2GO (version 5.2.5) [28, 29, 65]. To identify candidate genes, such as *DIR*, *IGS1*, and *PLR*, first, the homologous unigenes were searched against UniProt, TAIR, and Plant Metabolic Pathway (https://plantcyc.org) databases using BLASTX with a cutoff of *E*-value <1*E*-20. Then, these unigenes were curated based on their protein domain data by using InterProScan, compared with corresponding conserved protein domains via multiple-sequence alignment using MUSCLE (3.8.31) [66], and further analyzed by using COBALT [67] to visualize the conservation among sequences (https://www.ncbi.nlm.nih.gov/tools/cobalt). Phylogenetic analysis was performed using MEGA-X via the maximum likelihood-based method [68].

### Differential expression analysis

To examine differential expression patterns between red-colored fruit and yellow green leaf at 120 DAF for tissue-specific expression of transcripts, and between fruits collected at 40 (green-colored fruit berries) and 120 (deep red-colored fruit berries) DAF for transcriptome profiling during fruit development, respectively, RNA-Seq data derived from those four samples of Cheongsoon were analyzed. Clean RNA-Seq reads were mapped to the unigenes of *S. chinensis* using Bowtie 2 (version 2.4.4) [69]. Transcript abundance was estimated using RSEM (version 1.2.19) [70] as transcripts per million (TPM) values. Based on the resulting quantification results, the expression differences between fruit and leaf samples at 120 DAF, and between fruit samples collected at 40 and 120 DAF were analyzed using the TCC package (version 1.28.0) [71], respectively, with cutoffs of *q* < 0.05, an absolute fold-change ≥1.5, and an TPM ≥ 1 as the minimum average expression value between genes identified in the two samples. DEGs between fruit samples collected at 40 and 120 DAF were further annotated. GO enrichment analysis for DEGs that were specifically annotated with known genes in TAIR was performed by using DAVID (version 6.8) [72] according to an EASE score < 0.001. Genes categorized with GO-enriched terms were selected, and their expression patterns were visualized in a heatmap by using MeV (http://mev.tm4.org) with the Euclidean distance and complete linkage method. To investigate protein-protein interactions, genes categorized with specific GO terms were searched against STRING [73] with medium confidence (≥0.4). The network was further analyzed using Cytoscape (www.cytoscape.org).

## Supplementary Information

**Additional file 1: Table S1**. Summary of subreads generated by Iso-Seq.

**Additional file 2: Table S2**. Summary of RNA-Seq in *S. chinensis*.

**Additional file 3: Table S3**. Summary for circular consensus sequencing (CCS) reads.

**Additional file 4: Figure S1-S7**.

**Additional file 5: Table S4**. Phenotypic characteristics of *S. chinensis* samples used in the study.

**Additional file 6: Table S5**. Primers used for qRT-PCR validation.

## Data Availability

Sequencing data used in this study are available in the NCBI Sequence Read Archive (SRA) database under the following accession numbers: SAMN19471228 for PacBio Iso-Seq data (https://www.ncbi.nlm.nih.gov/biosample/SAMN19471228) and SAMN19471229 - SAMN19471233 (https://www.ncbi.nlm.nih.gov/biosample/SAMN19471229/; https://www.ncbi.nlm.nih.gov/biosample/SAMN19471230; https://www.ncbi.nlm.nih.gov/biosample/SAMN19471231; https://www.ncbi.nlm.nih.gov/biosample/SAMN19471232; https://www.ncbi.nlm.nih.gov/biosample/SAMN19471233) for Illumina RNA-Seq.

## Abbreviations

ABA: Abscisic acid
CEGMA: Core Eukaryotic Genes Mapping Approach
DAF: Day after flowering
DEG: Differentially expressed gene
DIR: Dirigent-like protein
EGS1: Eugenol synthase 1
GO: Gene ontology
HPLC: High-performance liquid chromatography
IGS1: Isoeugenol synthase 1
Iso-Seq: Isoform sequencing
JA: Jasmonic acid
KEGG: Kyoto Encyclopedia of Genes and Genomes
ORF: Open reading frame
PLR: Pinoresinol-lariciresinol reductase
RNA-Seq: RNA sequencing
SDR: Short-chain dehydrogenase/reductase
TE: Transposable element
TF: Transcription factor
