# Supplementary material for: Long-read transcriptome sequencing provides insight into lignan biosynthesis during fruit development in Schisandra chinensis
Source: BMC Genomics. 2022 Jan 8;23:17. doi: 10.1186/s12864-021-08253-2 (PMC8742460; doi:10.1186/s12864-021-08253-2)
Supplement: Supplementary file 1 — Additional file 1: Table S1. Summary of subreads generated by Iso-Seq. [file 12864_2021_8253_MOESM1_ESM.pdf]

Table S1. Summary of subreads generated by Iso-Seq

Sample ID	Species	Tissues	Sampling	Library size (kb)	Replicate	No. of subreads	Total bases
Sc_CS_Unigene_P_S_1-2kb_1	*Schisandra chinensis* cv. Cheongsoon	Pooled from leaves, roots, and fruits	- Fruits were sampled at 40 and 120 DAF*. - Leaves and roots were sampled at 28 DAF.	1~2 kb	1	832,695	1,497,092,426
Sc_CS_Unigene_P_S_1-2kb_2					2	822,969	1,488,974,100
Sc_CS_Unigene_P_S_1-2kb_3					3	834,873	1,505,411,982
Sc_CS_Unigene_P_S_2-3kb_1				2~3 kb	1	516,498	1,185,059,192
Sc_CS_Unigene_P_S_2-3kb_2					2	676,736	1,654,278,768
Sc_CS_Unigene_P_S_2-3kb_3					3	737,582	1,690,025,312
Sc_CS_Unigene_P_S_3-6kb_1				3~6 kb	1	823,775	1,781,193,440
Sc_CS_Unigene_P_S_3-6kb_2					2	856,604	1,823,713,588
Sc_CS_Unigene_P_S_3-6kb_3					3	758,081	1,678,586,774
Sc_CS_Unigene_P_S_>6kb_1				> 6 kb	1	945,597	1,705,975,962
Sc_CS_Unigene_P_S_>6kb_2					2	918,502	1,639,050,618
Sc_CS_Unigene_P_S_>6kb_3					3	733,938	1,598,206,863

*DAF, day after flowering
