# Supplementary material for: Long-read transcriptome sequencing provides insight into lignan biosynthesis during fruit development in Schisandra chinensis
Source: BMC Genomics. 2022 Jan 8;23:17. doi: 10.1186/s12864-021-08253-2 (PMC8742460; doi:10.1186/s12864-021-08253-2)
Supplement: Supplementary file 2 — Additional file 2: Table S2. Summary of RNA-Seq in S. chinensis. [file 12864_2021_8253_MOESM2_ESM.pdf]

Table S2. Summary of RNA-Seq in *S. chinensis*

Sample ID	Species	Tissues	Sampling	Replicate	No. of raw reads	No. of mapped reads	Mapping rate (%)
Sc_CS_FR_DAF40_1	*S. chinensis* cv. Cheongsoon	Fruit	40 DAF	1	71,971,122	68,919,546	95.76%
Sc_CS_FR_DAF40_2				2	64,364,590	61,976,663	96.29%
Sc_CS_FR_DAF40_3				3	87,435,052	84,051,315	96.13%
Sc_CS_FR_DAF120_1			120 DAF	1	62,094,036	59,939,372	96.53%
Sc_CS_FR_DAF120_2				2	70,047,440	66,397,968	94.79%
Sc_CS_FR_DAF120_3				3	81,832,554	78,837,482	96.34%
Sc_CS_LF_DAF120_1		Leaf	120 DAF	1	34,123,784	31,663,047	92.79%
Sc_CS_LF_DAF120_2				2	35,324,290	33,601,251	95.12%
Sc_CS_LF_DAF120_3				3	31,626,550	120,776,774	90.99%
Sc_CS_LF_DAF120_4				4	31,423,206	30,112,445	95.83%
Sc_CS_LF_DAF120_5				5	37,236,932	35,305,730	94.81%
Sc_SB_FR_DAF120_1	*S. chinensis* cv. Sobaeksan	Fruit	120 DAF	1	35,777,886	33,632,327	94.00%
Sc_SB_FR_DAF120_2				2	30,172,388	120,488,561	94.42%
Sc_SB_FR_DAF120_3				3	30,565,138	120,708,066	93.92%
Sc_SB_FR_DAF120_4				4	31,054,668	120,833,452	92.85%
Sc_SB_FR_DAF120_5				5	29,015,358	27,125,835	93.49%
Sc_SB_LF_DAF120_1		Leaf	120 DAF	1	32,504,046	31,297,842	96.29%
Sc_SB_LF_DAF120_2				2	31,641,596	29,916,166	94.55%
Sc_SB_LF_DAF120_3				3	30,711,906	29,334,896	95.52%
Sc_SB_LF_DAF120_4				4	120,724,826	26,965,384	93.87%
Sc_SB_LF_DAF120_5				5	31,740,064	297,312,095	93.68%

*DAF, day after flowering
