# Supplementary material for: Long-read transcriptome sequencing provides insight into lignan biosynthesis during fruit development in Schisandra chinensis
Source: BMC Genomics. 2022 Jan 8;23:17. doi: 10.1186/s12864-021-08253-2 (PMC8742460; doi:10.1186/s12864-021-08253-2)
Supplement: Supplementary file 3 — Additional file 3: Table S3. Summary for circular consensus sequencing (CCS) reads. [file 12864_2021_8253_MOESM3_ESM.pdf]

**Table S3**. Summary for circular consensus sequencing (CCS) reads

| | | 1-2 kb | 2-3 kb | 3-6 kb | > 6 kb |
|---|---|---|---|---|---|
| Classify | Reads of insert (CCS reads) (No.) | 221,926 | 261,685 | 304,839 | 292,537 |
| | 5' reads (No.) | 159,347 | 178,433 | 187,981 | 154,304 |
| | 3' reads (No.) | 156,094 | 185,429 | 186,328 | 173,513 |
| | Poly-A reads (No.) | 150,419 | 181,356 | 178,809 | 164,116 |
| | Filtration of CCS reads | | | | |
| | Filtered short reads (No.) | 9,392 | 7,622 | 10,080 | 12,119 |
| | Non-full-length reads (No.) | 88,933 | 112,982 | 165,881 | 177,571 |
| | Full-length chimeric reads (No.) | 1,101 | 1,079 | 788 | 680 |
| | Full-length non-chimeric reads (No.) * | 122,500 | 140,002 | 128,090 | 102,167 |
| | Average full-length non-chimeric read length (bp) | 1,728 | 2,689 | 2,896 | 2,724 |
| Cluster | Polished high-quality isoforms (No.) | 35,362 | 38,158 | 43,856 | 41,514 |
| | Polished low-quality isoforms (No.) | 15,935 | 29,873 | 35,366 | 33,285 |
| | Polished high-/low-quality isoforms (No.) * | 51,297 | 68,031 | 79,222 | 74,799 |
| | Average consensus isoforms read length (bp) | 1,768 | 2,682 | 2,813 | 2,930 |

*Reads used in the analysis
