# Supplementary material for: Long-read transcriptome sequencing provides insight into lignan biosynthesis during fruit development in Schisandra chinensis
Source: BMC Genomics. 2022 Jan 8;23:17. doi: 10.1186/s12864-021-08253-2 (PMC8742460; doi:10.1186/s12864-021-08253-2)
Supplement: Supplementary file 4 — Additional file 4: Figure S1-S7. [file 12864_2021_8253_MOESM4_ESM.pdf]

Supplementary Information

Figure S1. Number of unigenes containing the predicted ORFs.

Figure S2. Annotation of unigenes using InterPro (a) and UniProtKB (b) databases.

Figure S3. Contents of schisandrin in the fruit and leaf of *S. chinensis* by high-performance liquid chromatography (HPLC) profiling. CS, Cheongsoon; SB, Sobaeksan; F, fruit; L, leaf. Statistical significance was calculated with *t*-Test. The collected sample was pulverized after hot air drying at 65°C for 24 h and extracted using 100% methanol. Schisandrin was quantified using HPLC (Agilent 1200 series, C18 column (4.6 X 150 mm, 5 ml)). The mobile phase was acetonitrile and water in a gradient from 20:80 to 100:0, and the flow rate was 1.0 ml/min. Detection was conducted at 220 nm, and the column temperature was 35°C..

Figure S4. Differential expression of *CADs*, *IGS1s*, *DIR*, and *SILDs* at 40 and 120 DAF in *S. chinensis* fruit. The quantification of gene expression was estimated by using RSEM and represented as the normalized TPM value.

Figure S5. Mapping of differentially expressed genes (DEGs) that were upregulated at 120 DAF in *S. chinensis* fruit to a KEGG pathway map of glycolysis/gluconeogenesis. Copyright permission was granted by Kanehisa Laboratories to publish the KEGG pathway map image (00010) under the CC BY 4.0 open access license.

Figure S6. qRT-PCR validation for *CADs*, *IGS1s*, and *DIR* in *S. chinensis*. Relative expression level of *CADs* (unigenes: KSC_ISO_007296, _012053, _040169, and _069272), *IGS1s* (KSC_ISO_083521 and _090301), and *DIR* (KSC_ISO_008873) between 40 DAF and 120 DAF in *S. chinensis* was analyzed by qRT-PCR (n=3, error bars indicate standard error). Relative gene expression levels between qRT-PCR and RNA-Seq were compared. Gene specific primers used for qRT-PCR are listed in Table S5. qRT-PCR was performed with a Quant Studio 3 (Applied Biosystems) instrument using SYBR Green Real-time PCR Master Mix (Applied Biosystems) based on the manufacturer’s instructions.

Figure S7. An overview of lignan biosynthesis in plant.

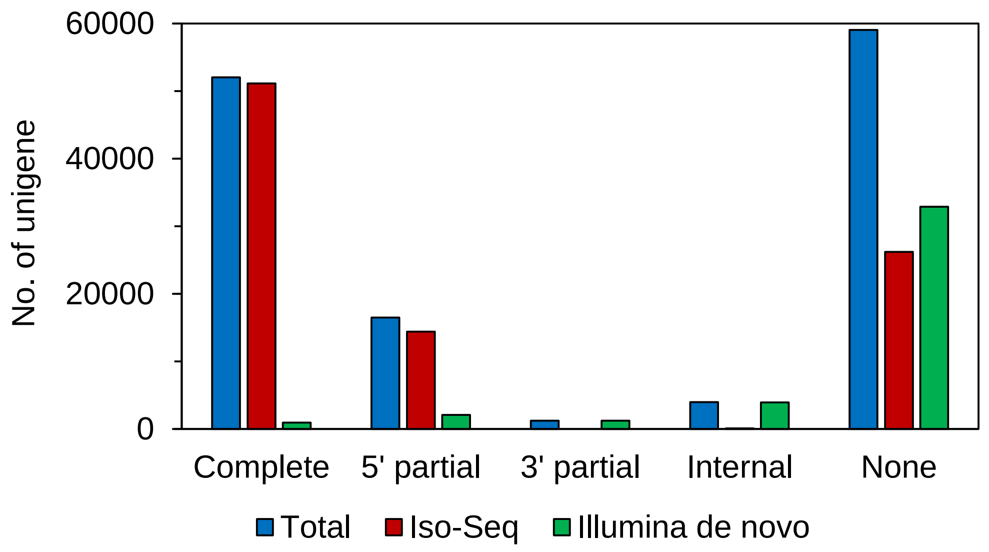

Figure S1. Number of unigenes containing the predicted ORFs.

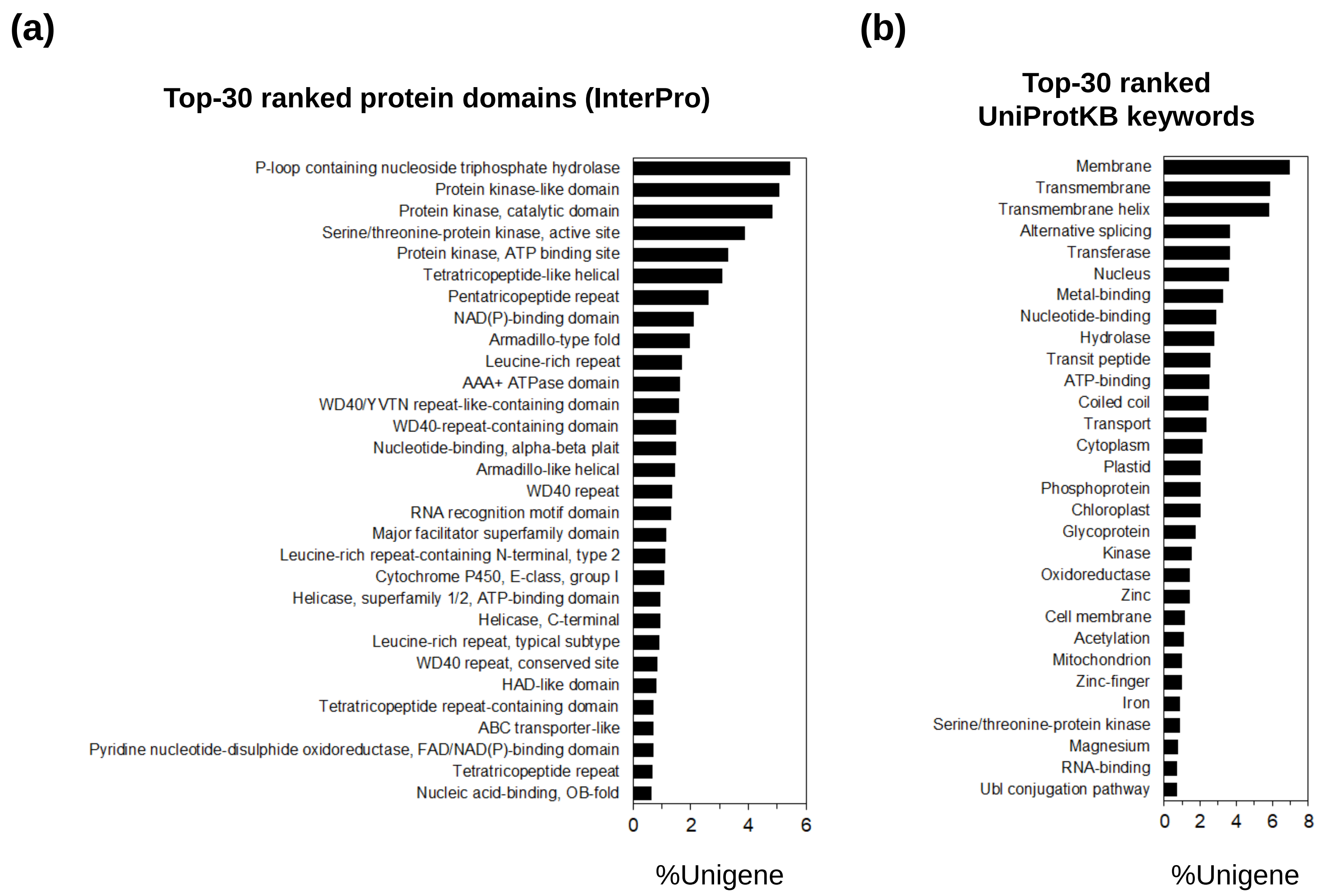

Figure S2. Annotation of unigenes using InterPro (a) and UniProtKB (b) databases.

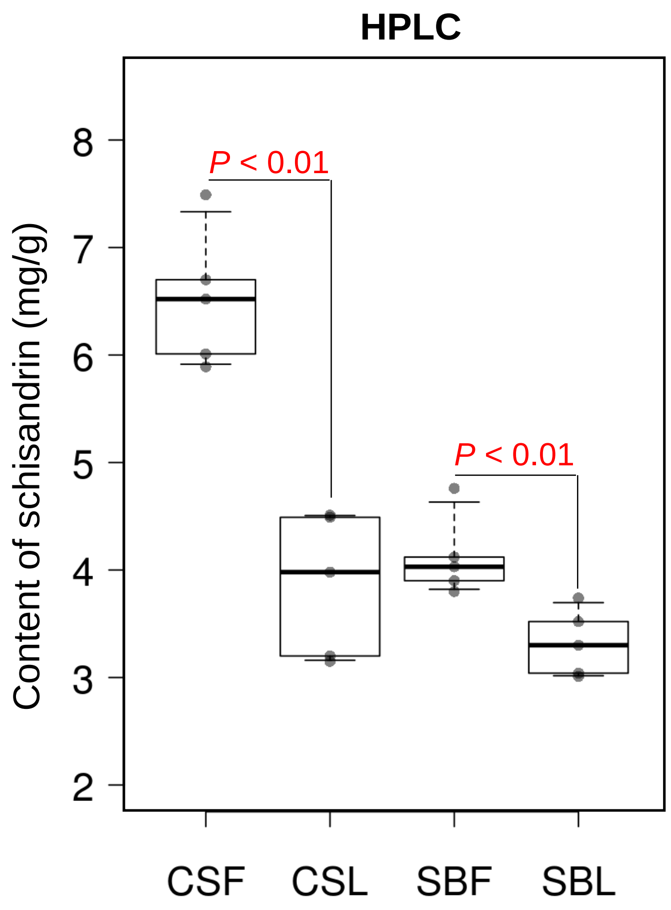

Figure S3. Contents of schisandrin in the fruit and leaf of *S. chinensis* by high-performance liquid chromatography (HPLC) profiling. CS, Cheongsoon; SB, Sobaeksan; F, fruit; L, leaf. Statistical significance was calculated with *t*-Test. The collected sample was pulverized after hot air drying at 65°C for 24 h and extracted using 100% methanol. Schisandrin was quantified using HPLC (Agilent 1200 series, C18 column (4.6 X 150 mm, 5 ml)). The mobile phase was acetonitrile and water in a gradient from 20:80 to 100:0, and the flow rate was 1.0 ml/min. Detection was conducted at 220 nm, and the column temperature was 35°C.

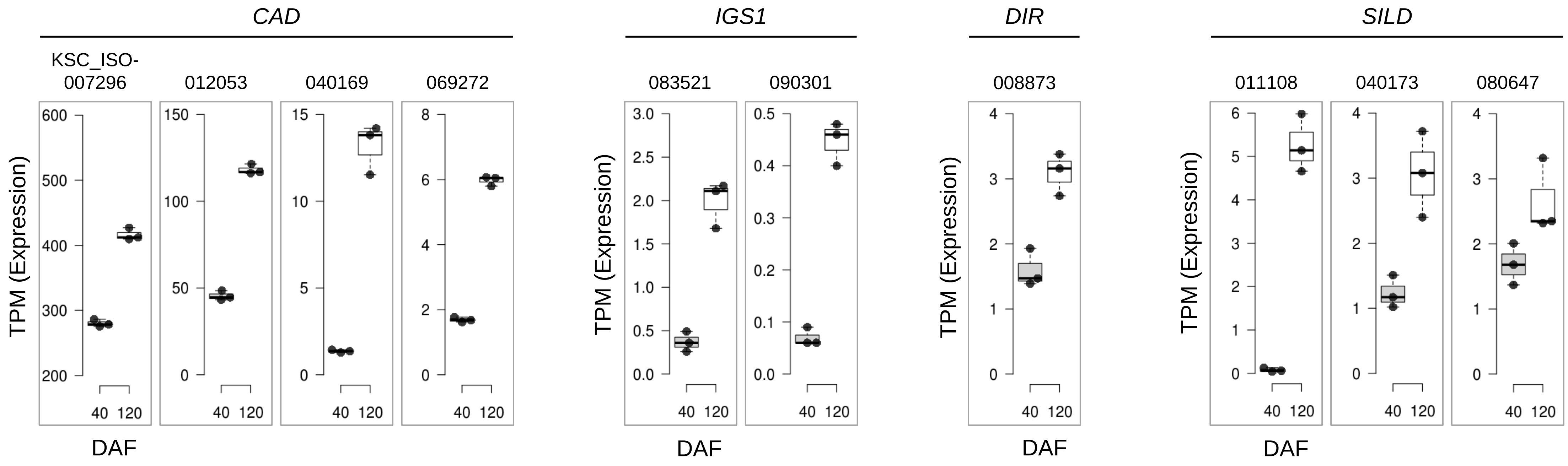

q-value	1.44×10^{-24}	1.07×10^{-91}	1.79×10^{-122}	3.72×10^{-29}	5.74×10^{-10}	7.22×10^{-5}	0.0014	0.04	1.47×10^{-45}	0.0005
Fold change (Log_2 - scale)	0.65	1.46	3.36	1.90	2.49	2.68	1.02	1.46	6.16	1.38

Figure S4. Differential expression of *CADs*, *IGS1s*, *DIR*, and *SILDs* at 40 and 120 DAF in *S. chinensis* fruit. The quantification of gene expression was estimated by using RSEM and represented as the normalized TPM value.

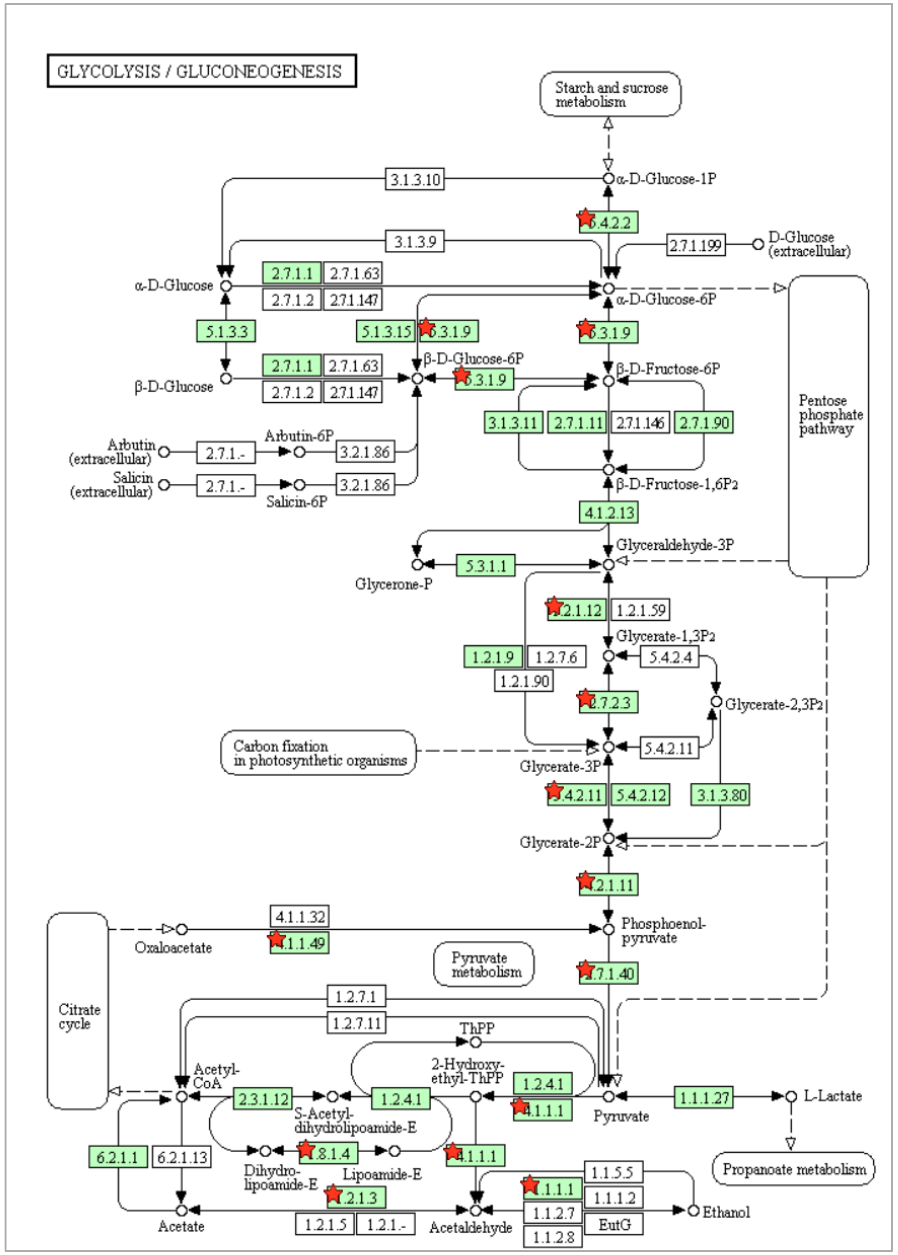

Figure S5. Mapping of differentially expressed genes (DEGs) that were upregulated at 120 DAF in *S. chinensis* fruit to a KEGG pathway map of glycolysis/ gluconeogenesis. Copyright permission was granted by Kanehisa Laboratories to publish the KEGG pathway map image (00010) under the CC BY 4.0 open access license.

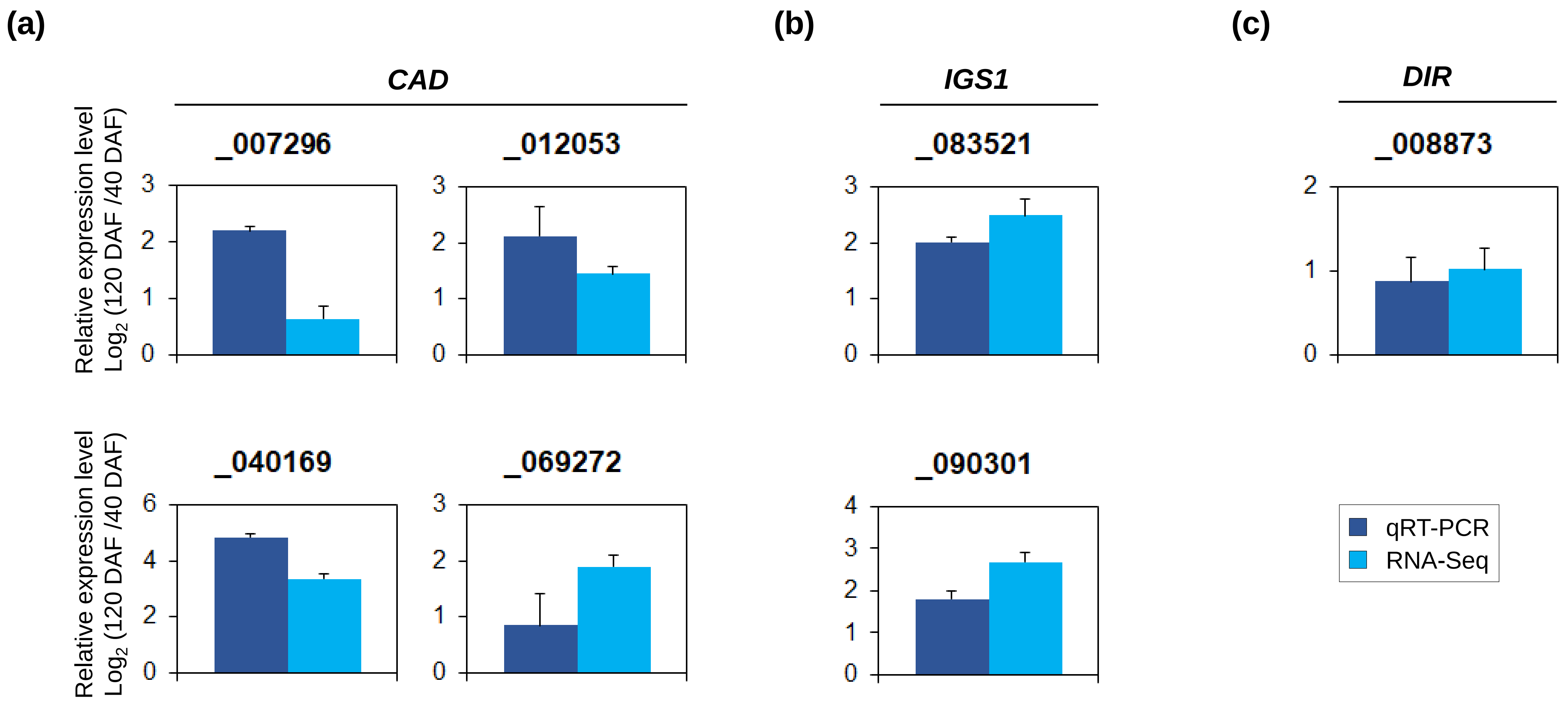

Figure S6. qRT-PCR validation for *CADs*, *IGS1s*, and *DIR* in *S. chinensis*. Relative expression level of *CADs* (unigenes: KSC_ISO_007296, _012053, _040169, and _069272), *IGS1s* (KSC_ISO_083521 and _090301), and *DIR* (KSC_ISO_008873) between 40 DAF and 120 DAF in *S. chinensis* was analyzed by qRT-PCR (n=3, error bars indicate standard error). Relative gene expression levels between qRT-PCR and RNA-Seq were compared. Gene specific primers used for qRT-PCR are listed in **Table S5**. qRT-PCR was performed with a Quant Studio 3 (Applied Biosystems) instrument using SYBR Green Real-time PCR Master Mix (Applied Biosystems) based on the manufacturer’s instructions.

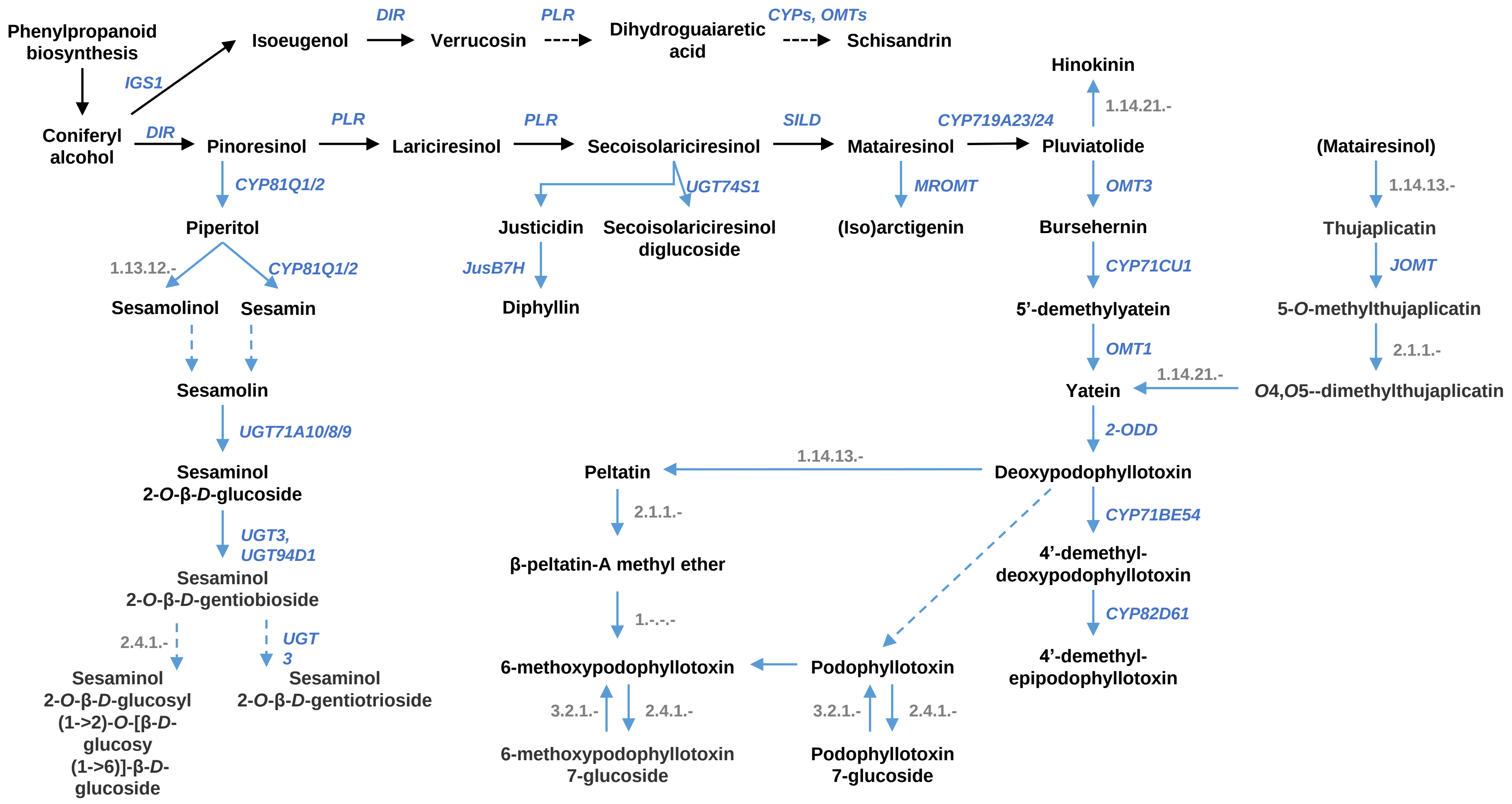

Figure S7. An overview of lignan biosynthesis in plant.
