# Supplementary material for: Long-read transcriptome sequencing provides insight into lignan biosynthesis during fruit development in Schisandra chinensis
Source: BMC Genomics. 2022 Jan 8;23:17. doi: 10.1186/s12864-021-08253-2 (PMC8742460; doi:10.1186/s12864-021-08253-2)
Supplement: Supplementary file 5 — Additional file 5: Table S4. Phenotypic characteristics of S. chinensis samples used in the study. [file 12864_2021_8253_MOESM5_ESM.pdf]

Table S4. Phenotypic characteristics of *S. chinensis* samples used in the study

Sample	Cultivar or wild type	Leaf		Petal color	Fruit		Fruit bunch			Weight (g) / 100 berries	%Flower drop	%Infected by brown leaf spot / powdery mildew
		Shape	Color		Shape	Color	Length (cm)	Width (cm)	Weight (g)			
Cheongsoon	Cultivar	Oval	Yellow green	Light red	Circle	Deep red	5.5	2.4	10.1	66.6	1.9	~5% / ~20%
Sobaeksan	Wild type (collected from Mungyeong, Republic of Korea)	Long oval	Green	White	Circle	Red	5.4	2.0	9.4	47.0	11.0	~20% / ~30%
