# Supplementary material for: Long-read transcriptome sequencing provides insight into lignan biosynthesis during fruit development in Schisandra chinensis
Source: BMC Genomics. 2022 Jan 8;23:17. doi: 10.1186/s12864-021-08253-2 (PMC8742460; doi:10.1186/s12864-021-08253-2)
Supplement: Supplementary file 6 — Additional file 6: Table S5. Primers used for qRT-PCR validation. [file 12864_2021_8253_MOESM6_ESM.pdf]

Table S5. Primers used for qRT-PCR validation

Unigene ID	Gene	Primer sequence	
		Forward	Reverse
KSC_ISO_007296	*CAD*	TACCTGGATGGAAAGCCAAC	AACCCAAAATCCCACCTTTC
KSC_ISO_012053		TTATGCTTGGGAGGAAATCG	CAGCCACATCAACCACAAAC
KSC_ISO_040169		AGCAAGCAGCTCCTCTTCTG	GCCCAAGTGTTCAATTGCTT
KSC_ISO_069272		AATGGTCCCAGGACATGAAG	GTTGGCTTTCCATCTGGGTA
KSC_ISO_083521	*IGS1*	CCTGCAGTTCAAGGGATGTT	TGGCTTTCCCTGTTCCATAG
KSC_ISO_090301		TGGTTTGGTTTGGTTTGGAT	CTGTCTTATCCGCCCACATT
KSC_ISO_008873	*DIR*	GCAGAAGATCAGCCACTTCC	AATTCATGGCCATCAGAAGC
